# Tau‐induced upregulation of C/EBPβ‐TRPC1‐SOCE signaling aggravates tauopathies: A vicious cycle in Alzheimer neurodegeneration

**DOI:** 10.1111/acel.13209

**Published:** 2020-08-20

**Authors:** Jinwang Ye, Ying Yin, Yaling Yin, Huaqiu Zhang, Huali Wan, Lu Wang, Yue Zuo, Di Gao, Mengzhu Li, Jun Li, Yanchao Liu, Dan Ke, Jian‐Zhi Wang

**Affiliations:** ^1^ Key Laboratory of Ministry of Education of China for Neurological Disorders Tongji Medical College Huazhong University of Science and Technology Wuhan China; ^2^ Department of Physiology and Neurobiology School of Basic Medical Sciences Xinxiang Medical University Xinxiang China; ^3^ Department of Neurosurgery Key Laboratory of Ministry of Education of China for Neurological Disorders Tongji Hospital Huazhong University of Science and Technology Wuhan China; ^4^ Department of Neurosurgery The Central Hospital of Wuhan Tongji Medical College Huazhong University of Science and Technology Wuhan China; ^5^ Co‐innovation Center of Neurodegeneration Nantong University Nantong China

## Abstract

Intracellular accumulating of the hyperphosphorylated tau plays a pivotal role in neurodegeneration of Alzheimer disease (AD), but the mechanisms underlying the gradually aggravated tau hyperphosphorylation remain elusive. Here, we show that increasing intracellular tau could upregulate mRNA and protein levels of TRPC1 (transient receptor potential channel 1) with an activated store‐operated calcium entry (SOCE), an increased intraneuronal steady‐state [Ca^2+^]_i_, an enhanced endoplasmic reticulum (ER) stress, an imbalanced protein kinases and phosphatase, and an aggravated tauopathy. Furthermore, overexpressing TRPC1 induced ER stress, kinases‐phosphatase imbalance, tau hyperphosphorylation and cognitive deficits in cultured neurons and mice, while pharmacological inhibiting or knockout TRPC1 attenuated the hTau‐induced deregulations in SOCE, ER homeostasis, kinases‐phosphatase balance, and tau phosphorylation level with improved synaptic and cognitive functions. Finally, an increased CCAAT‐enhancer‐binding protein (C/EBPβ) activity was observed in hTau‐overexpressing cells and the hippocampus of the AD patients, while downregulating C/EBPβ by siRNA abolished the hTau‐induced TRPC1 upregulation. These data reveal that increasing intracellular tau can upregulate C/EBPβ‐TRPC1‐SOCE signaling and thus disrupt phosphorylating system, which together aggravates tau pathologies leading to a chronic neurodegeneration.

## Graphical abstract

Increasing tau accumulation leads to hyperactivated SOCE, ER stress, and kinases‐phosphatase imbalance through C/EBPβ‐mediated upregulation of TRPC1, which thereby induces persistent and aggravated tau hyperphosphorylation and cognitive deficits in cultured neurons or mice, while pharmacological inhibiting or knockout TRPC1 attenuated the hTau‐induced dysregulations in SOCE, ER homeostasis, kinases‐phosphatase balance, and tau phosphorylation level with improved synaptic and cognitive functions.

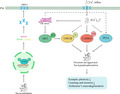

## 1 INTRODUCTION

Intracellular accumulation of the hyperphosphorylated tau forming paired helical filaments (PHF)/neurofibrillary tangles (NFTs) is hallmark of Alzheimer disease (AD) and the related tauopathies (Grundke‐Iqbal et al., 1986). The axonal PHF‐tau pathology in hippocampal pathways is critical for the clinical expression of dementia and may constitute an anatomical substrate of clinically verifiable memory dysfunction in AD (Thal et al., 2000). Elevated plasma total tau levels are associated with cognitive decline and risk of mild cognitive impairment (Mielke et al., 2017), and dendritic function of tau mediates amyloid‐β toxicity (Ittner et al., 2010). Dietary salt promotes tau phosphorylation and tau‐null blocked salt‐induced cognitive impairment (Faraco et al., 2019). However, it is not known why tau proteins can be persistently hyperphosphorylated and gradually aggregated during AD progression.

AD‐like abnormal tau hyperphosphorylation is believed to be caused by disturbance of the corresponding tau candidate protein kinases and protein phosphatases. More than ten serine/threonine protein kinases have been shown to phosphorylate tau at several specific pathological sites, including ERK1/2, CDK5, GSK3β, CaMKII, PKA, PKC, and CK2 (Wang & Liu, 2008). Among them, GSK3β is the most implicated in tau hyperphosphorylation in the AD brain. Moreover, GSK3β is the first identified tau kinase and participates in both tau and amyloid pathologies in AD, which is also regarded as a molecular link between the two major histopathological hallmarks of the disease (Avila, Wandosell, & Hernandez, 2010; Ishiguro, Omori, et al., 1992; Ishiguro, Takamatsu, et al., 1992). At least five protein phosphatases are highly expressed in the mammalian brains, including PP1, PP2A, PP2B, PP2C, and PP5, and all of them, except PP2C, are shown to dephosphorylate tau in vitro and possibly in vivo as well (Gong, Liu, Grundke‐Iqbal, & Iqbal, 2005). It is widely accepted that PP2A is the major tau phosphatase, accounting for approximately 70% of tau phosphatase activity in human brain (Liu, Grundke‐Iqbal, Iqbal, & Gong, 2005). Decreased PP2A activity has been evidenced in the frontal and temporal cortices of AD patients (Sontag et al., 2004), which promotes tau hyperphosphorylation, and subsequent NFTs formation and synaptic degeneration (Shentu et al., 2018; Sun et al., 2012).

Store‐operated Ca^2+^ entry (SOCE) plays a vital role in central nervous system, and deregulation of SOCE causes neural inflammation, synapse impairments, and neuron death (Hao et al., 2014; Sun et al., 2014; Szteyn, Gomez, Berg, & Jeske, 2015). The molecular components of SOCE contain stromal interaction molecules (STIM1/2), ORAIs (ORAI1/2/3), and transient receptor potential channels (TRPC1‐7). Upon depletion of the Ca^2+^ store in endoplasmic reticulum (ER), STIM1/2 sense ER Ca^2+^ reduction and thus oligomerize, and subsequently translocate from ER‐like sites to the plasma membrane and interact with calcium‐conducting channels (ORAIs and TRPCs) to induce Ca2+ influx and store refilling (Zhang et al., 2005, 2016). Among them, TRPC1 and STIM1/2 have been identified in neurodegeneration. For instance, the neuronal SOCE enhancement with elevated STIM2 expression was seen in medium spiny neurons (MSNs) of YAC128 Huntington's disease (HD) mice, and inhibition of SOCE or knockdown of STIM2 improved dendritic spines deficiency in these mice (Wu et al., 2016). In Parkinson disease (PD), store depletion and subsequent activation of TRPC1 via STIM1 inhibits the frequency and amplitude of the rhythmic activity in dopaminergic neurons and protects dopaminergic neurons from death, application of PD‐mimicking neurotoxins induces downregulation of TRPC1, overexpression of TRPC1 protects cells against neurotoxin‐mediated cytotoxicity (Sun et al., 2017, 2018). In AD, familial AD‐associated presenilin 1 mutants promote γ‐secretase cleavage of STIM1 to impair SOCE (Tong et al., 2016). Level of STIM2 was reduced in hippocampal neurons from APP‐KI and PS1‐M146V‐KI mouse model and cortical samples of AD patients, which results in mushroom spine loss through STIM2‐regulated synaptic SOCE reduction, and overexpression of STIM2 restored the hippocampal mushroom spine deficiency in these AD mice models (Sun et al., 2014) (Pchitskaya, Popugaeva, & Bezprozvanny, 2018; Zhang et al., 2015). However, whether SOCE plays a role in AD‐related tauopathy remain poorly understood.

In the current study, we overexpressed full‐length wild‐type human tau (termed hTau) both in vitro and in vivo to mimic the early‐stage tau pathology seen in sporadic AD and studied how the increased intracellular hTau in turn aggravates tau pathologies and elicits learning and memory deficits. We found that overexpressing hTau upregulated C/EBPβ and its downstream TRPC1‐dependent SOCE with a simultaneous ER stress and imbalance of protein kinases and phosphatase. Inhibiting or knocking out TRPC1 attenuated tau pathologies both in vivo and in vitro with improvement of learning and memory in mice, whereas overexpressing TRPC1 induces ER stress and deregulates protein kinases and phosphatase with aggravated tau pathologies and memory deficits. Furthermore, downregulating C/EBPβ attenuated hTau‐induced TRPC1 elevation with attenuation of ER stress and tau pathologies.

## 2 RESULTS

### 2.1 Overexpressing hTau enhances TRPC1‐dependent SOCE signaling with ER stress and dysregulation of protein kinases and phosphatase

**FIGURE 1:**
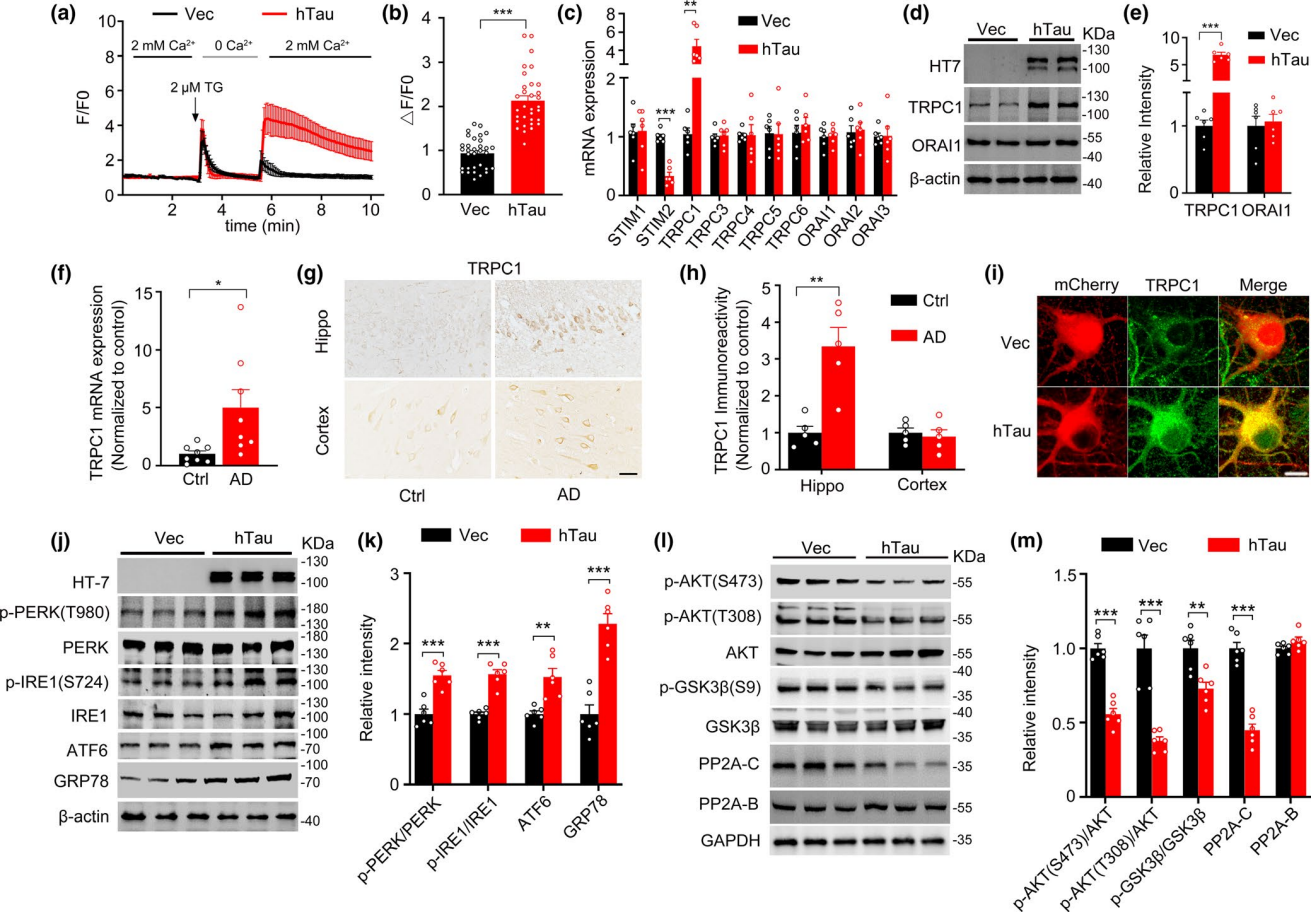
Overexpressing hTau increases TRPC1‐dependent SOCE with ER stress and dysregulation of protein kinases and phosphatase. (a, b) Overexpressing hTau increases SOCE. The primary hippocampal neurons (5 *div*) were infected with lenti‐syn‐hTau‐mCherry or the empty vector and cultured for another 7 days, and then, the neurons were loaded with Fluo3‐AM for Ca^2+^ imaging. The representative curves of the time scan of intracellular Ca^2+^ signals by following store‐depletion protocol (a) and the quantification of the amplitude of Ca^2+^ influx (b). *N* = 32 for each group, unpaired Student's *t*‐test. (c) Overexpressing hTau increases TRPC1 mRNA and decreases STIM2 with no significant effects on other candidates measured by qPCR. *N* = 6 for each group, unpaired Student's *t*‐test. (d, e) Overexpressing hTau (HT7) increases TRPC1 protein level without changing ORAI1 and β‐actin. *N* = 6 for each group, unpaired Student's *t*‐test. (f‐h) The increased TRPC1 mRNA (*N* = 8) and protein (*N* = 5) levels detected in the hippocampi of AD patients compared to the age‐matched controls (Ctrl) by qPCR or immunohistochemistry. Unpaired Student's *t*‐test. (i) Representative images show TRPC1 elevation and its co‐staining with hTau in hTau‐overexpressing neurons. (j, k) Overexpressing hTau in cultured hippocampal neurons induces activation of ER stress pathways, demonstrated by elevated p‐PERK, p‐IRE1, ATF6, and GRP78, measured by Western blotting. β‐actin was used as a loading control. *N* = 6 for each group, unpaired Student's *t*‐test. (l, m) Overexpressing hTau induces inhibition of AKT and PP2A and activation of GSK‐3β measured by Western blotting. *N* = 6 for each group, unpaired Student's *t*‐test. Data were expressed as mean ±SD for (a) and mean ± SEM for (b–m), *p < 0.05, **p < 0.01, ***p < 0.001

Overexpressing hTau increased intraneuronal basal [Ca^2+^]_i_ (Yin, Gao, et al., 2016; Yin, Wang, et al., 2016). To explore the role of store‐operated Ca^2+^ entry (SOCE) in hTau‐induced Ca^2+^ dyshomeostasis, we overexpressed hTau (lenti‐syn‐hTau‐mCherry, termed as hTau) or the empty vector (lenti‐syn‐mCherry) in primary hippocampal neurons cultured 5 days in vitro (*div*). After 7 days, the neurons were loaded with Fluo3‐AM dye and the amplitude of SOCE was measured using a confocal microscopy. After depletion of ER (endoplasmic reticulum) Ca^2+^ store with 2 μM thapsigargin (TG, an ER Ca^2+^‐ATPase inhibitor), we found that overexpressing hTau significantly increased SOCE‐Ca^2+^ influx compared with the control neurons (Figure 1a,b). Previous studies indicate that transient receptor potential channels (TRPC1‐TRPC7), stromal interaction molecules (STIM1/2), and ORAI channels (ORAI1‐ORAI3) are key components of SOCE pathway (Liao et al., 2008). To identify the molecules responsible for the hTau‐enhanced SOCE, we performed RT‐qPCR to measure the mRNA levels of TRPC1, TRPC3‐TRPC7, STIM1/2, and ORAI1‐ORAI3. We found that overexpressing hTau dramatically increased TRPC1 mRNA with a decreased STIM2 mRNA and unchanged other candidates (Figure 1c). Further studies demonstrated that overexpressing hTau also increased protein level of TRPC1 without changing ORAI1 (Figure 1d,e). The increased mRNA and protein levels of TRPC1 were also detected in the hippocampal extracts of human AD brains compared with the age‐matched controls (Figure 1f–h), and colocalization of TRPC1 with GFP‐tagged hTau was detected in cultured primary hippocampal neurons (Figure 1i). TRPC2 is a pseudogene in humans and limitedly expressed in other species (Cheng, Ong, Liu, & Ambudkar, 2013).

As SOCE plays an important role in increasing intracellular [Ca^2+^] and ER Ca^2+^ refilling, we tested components of ER stress. The results showed that overexpressing hTau increased levels of phospho‐PERK (Thr980) (protein kinase R‐like ER kinase), phospho‐IRE1 (Ser724) (inositol‐requiring enzyme 1), ATF6 (activating transcription factor 6), and GRP78 (glucose regulated protein 78, an ER chaperon protein) (Figure 1j,k), suggesting that overexpressing hTau induces ER stress.

ER stress activates AKT/GSK3β signaling, which can robustly phosphorylate tau (Yuan et al., 2015). Therefore, we measured the expression level or the activity‐dependent changes of the related protein kinases and protein phosphatase. Overexpressing hTau decreased the levels of phospho‐AKT (protein kinase B, PKB) at Ser473 and Thr308, and phospho‐GSK3β at Ser9 (Figure 1l,m), suggesting inhibition of AKT and activation of GSK3β by hTau. We also measured PP2A, the most active tau phosphatase that regulates its phosphorylation at multiple pathological sites (Liu et al., 2005). Overexpressing hTau also decreased the level of PP2A‐C (PP2A catalytic subunit C) without changing PP2A‐B (PP2A regulatory subunit B) (Figure 1l,m), suggesting inhibition of PP2A by hTau. These data together indicate that increasing intracellular hTau induces TRPC1‐dependent SOCE‐mediated ER stress and dysregulates protein kinases and phosphatase, which can aggravate tau pathologies.

### 2.2 Upregulating TRPC1 aggravates tau pathologies involving ER stress and dysregulation of AKT/GSK3β and PP2A both* in vitro* and *in vivo*

**FIGURE 2:**
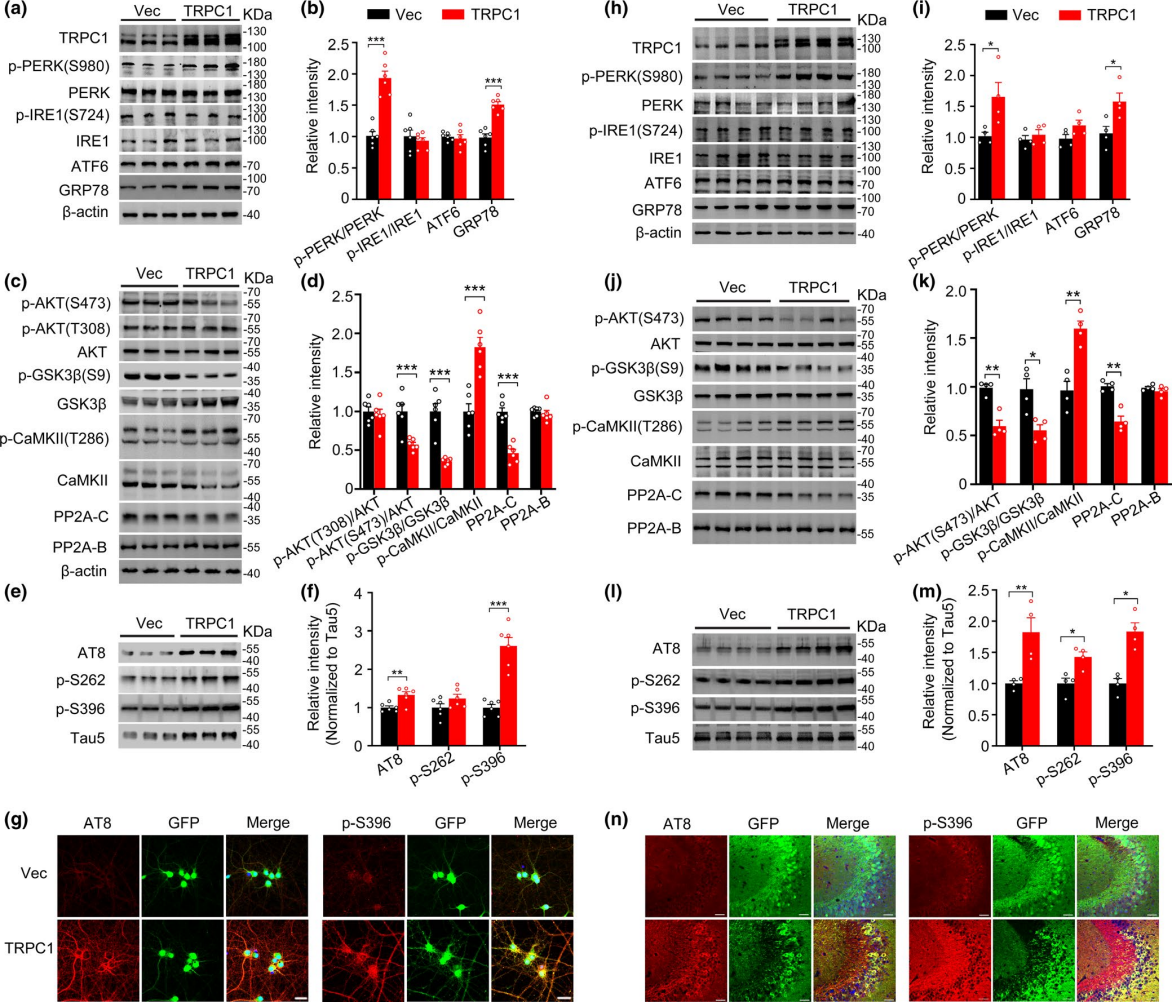
Upregulating TRPC1 induces ER stress and dysregulation of protein kinases and phosphatase with tau hyperphosphorylation both in vitro and in vivo. (a, b) Overexpressing TRPC1 (lenti‐syn‐TRPC1‐eGFP) induces ER stress in primary cultured hippocampal neurons (5 + 7 *div*) measured by Western blotting. The lenti‐syn‐eGFP was expressed as control. *N* = 6 for each group, unpaired Student's *t*‐test. (c, d) Overexpressing TRPC1 dysregulates protein kinases and phosphatase measured by Western blotting. *N* = 6 for each group, unpaired Student's *t*‐test. (e–g) Overexpressing TRPC1 increases tau phosphorylation at AT8, p‐S262, p‐S396 epitopes with an increased total tau (Tau5) measured by Western blotting and immunofluorescence staining using eGFP‐unfused lenti‐syn‐TRPC1‐T2A‐eGFP and lenti‐syn‐T2A‐eGFP. *N* = 6 for each group, unpaired Student's *t*‐test; scale bar = 20 μm. (h, i) Overexpressing TRPC1 induces ER stress in mice. AAV‐syn‐TRPC1‐eGFP or the empty vector was infused stereotaxically into the hippocampal CA3 of 2‐month‐old mice for one month, and then, the mice were sacrificed and the hippocampal CA3 were dissected for Western blotting analysis of (p‐)PERK, (p‐)IRE1, (p‐)ATF6, and GRP78. *N* = 4 for each group, unpaired Student's *t*‐test. (j, k) Overexpressing TRPC1 dysregulates protein kinases and phosphatase in vivo measured by Western blotting. *N* = 4 for each group, unpaired Student's *t*‐test. (l–n) Overexpressing TRPC1 increases tau phosphorylation at AT8, p‐S262, p‐S396 epitopes in vivo measured by Western blotting (l, m) and immunofluorescence staining (n). *N* = 4 for each group, unpaired Student's *t*‐test; scale bar = 50 μm. Data were expressed as mean ± SEM, *p < 0.05, **p < 0.01, ***p < 0.001

To validate the role of TRPC1‐dependent SOCE in hTau‐induced kinases and phosphatase dysregulation, we infected primary hippocampal neurons (*5 div*) with lenti‐syn‐TRPC1‐eGFP or lenti‐syn‐eGFP for 7 days. Overexpressing TRPC1 increased levels of GRP78 and p‐PERK (Ser980) with unchanged IRE‐1 and ATF6 (Figure 2a,b) with decreased levels of p‐AKT (Ser493) and p‐GSK3β (Ser9) (Figure 2c,d), and as well as a decreased PP2A‐C protein expression without changing PP2A‐B (Figure 2c,d). CaMKII is a major kinase targeting Ser262 phosphorylation of tau proteins and the increased intracellular [Ca^2+^] can activate CaMKII (Sironi et al., 1998). We also observed that overexpressing TRPC1 increased the level of p‐CaMKII (Thr286) (Figure 2c,d). These data confirm the role of TRPC1 upregulation in ER stress and the dysregulation of protein kinases and phosphatase. Simultaneously, the phosphorylation of tau at AT8 and p‐S396 epitopes (normalized to the total tau, tau5) was also increased in TRPC1‐overexpressing neurons (Figure 2e,f). The TRPC1‐induced tau hyperphosphorylation was detected in both the cell bodies and the processes by immunofluorescence staining on primary hippocampal neurons (Figure 2g). These in vitro data indicate that the hTau‐induced upregulation of TRPC1 can in turn aggravate tau pathologies by inducing ER stress and disrupting kinases and phosphatase.

To verify the in vivo role of TRPC1 upregulation, we overexpressed TRPC1 by stereotaxically injecting AAV‐syn‐TRPC1‐eGFP or AAV‐syn‐eGFP into the dorsal hippocampal CA3 of 2‐month‐old wild‐type mice. After one month, the hippocampal CA3 was carefully dissected and the extracts were prepared for Western blotting. Consistent with the in vitro results, overexpressing TRPC1 in mouse hippocampi also remarkably increased the levels of GRP78 and p‐PERK (Thr980) with minor effect on IRE1 and ATF6 (Figure 2h,i). Simultaneously, the levels of p‐AKT (Ser473), p‐GSK3β (Ser9), and PP2A‐C decreased, and level of p‐CaMKII (Thr286) increased after TRPC1 overexpression (Figure 2j,k). The increased levels of phosphorylated tau were also observed after TRPC1 overexpression (Figure 2l,m). The phosphorylated tau at AT8 and p‐S396 epitopes was co‐stained with TRPC1‐GFP‐positive neurons (Figure 2n). These in vivo data further confirm the role of hTau‐induced upregulation of TRPC1 in aggravating tau pathologies with the mechanisms involving ER stress signaling.

### 2.3 Upregulating TRPC1 induces learning and memory deficits in mice

**FIGURE 3:**
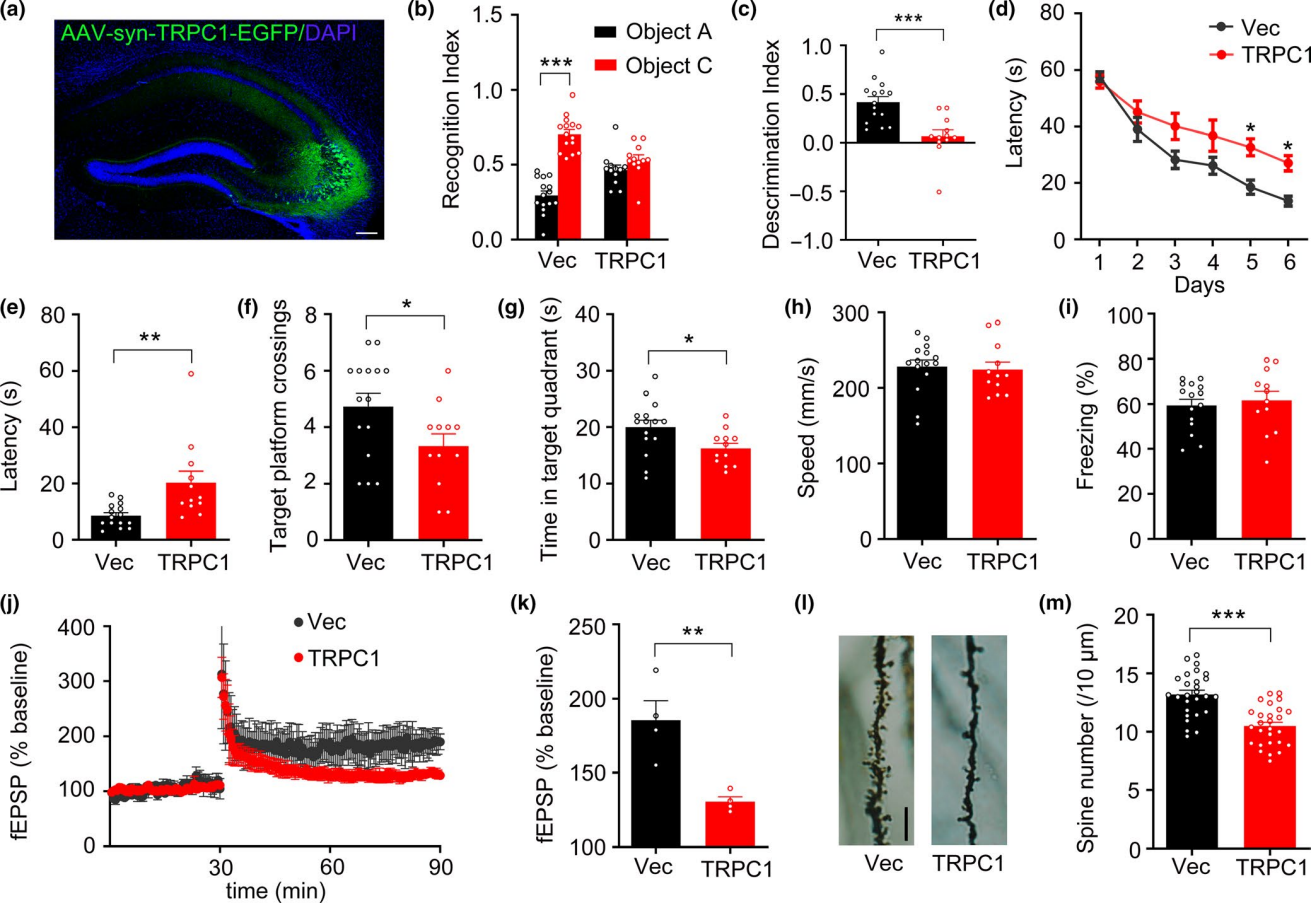
Upregulating TRPC1 induces learning and memory deficits in mice. (a) The representative image shows expression of TRPC1. AAV‐syn‐TRPC1‐eGFP or AAv‐syn‐eGFP was infused into the hippocampal CA3 subset of 2‐month‐old wild‐type (WT) C57BL/6 mice, after one month, the expression of TRPC1 in the infused site was confirmed by direct fluorescence imaging. Scale bar, 100 μm. (b, c) Overexpressing TRPC1 induces memory deficit measured by novel object recognition (NOR) test. The recognition index (b) and the discrimination index (c) recorded at 24 hr after training were analyzed. *N* = 15 for vector group and *N* = 12 for TRPC1 group, unpaired Student's *t*‐test. (d–h) Overexpressing TRPC1 induces spatial learning and memory deficits measured by Morris water maze (MWM). The spatial learning deficit was shown by the increased latency to find the hidden platform at day 5 and day 6 during 6 days training (d), while the memory deficit was shown by the increased latency to reach the platform site (e), reduced target platform crossings (f), and decreased time spent in the target zone (g) measured at day 8 by removed the hidden platform. The swimming speed was comparable between the two groups (h). *N* = 15 for vector group and *N* = 12 for TRPC1 group, unpaired Student's *t*‐test. (i) Overexpressing TRPC1 has minor effect on contextual memory. Analysis of the total freezing time in 3 min recorded during the contextual fear conditioning test. *N* = 15 for vector group and *N* = 12 for TRPC1 group, unpaired Student's *t*‐test. (j, k) Overexpressing TRPC1 impairs hippocampal LTP shown by the decreased fEPSP slope induced by applying 3 trains of high‐frequency stimulation (HFS) and normalized to the baseline. *N* = 4 for each group, unpaired Student's *t*‐test. (l, m) Overexpressing TRPC1 induces spine loss in hippocampal CA3 measured by Golgi staining. The spine number per 10 μm of dendritic length was analyzed, scale bar = 10 μm. *N* = 30 neurons from 3 mice for each group, unpaired Student's *t*‐test. Data were expressed as mean ± SEM, *p < 0.05, **p < 0.01, ***p < 0.001

The expression of AAV‐syn‐TRPC1‐eGFP was also confirmed by fluorescence imaging at 1 month after the injection (Figure 3a), and then, the effect of TRPC1 on learning and memory of mice was verified by novel object recognition (NOR) and Morris water maze (MWM). In NOR test, the recognition index was equivalent between control and TRPC1‐overexpressing mice in the training trial. Twenty‐four hours after the training trial, the recognition index (Figure 3b) and discrimination index (Figure 3c) to the novel object was significantly decreased in the TRPC1 mice. In MWM test, we observed that TRPC1 mice showed impaired spatial learning ability, indicated by the significantly longer latency at the last two days in the training trial (Figure 3d). In the memory test at day 8 by removing the platform, TRPC1 mice exhibited marked memory deficit, manifested by increased latency to find the platform (Figure 3e), decreased target platform crossings (Figure 3f) and time spend in target quadrant (Figure 3g), no significant difference in swimming speed was seen between the two groups (Figure 3h). In the contextual fear conditioning test, TRPC1 mice showed comparable contextual memory with control mice (Figure 3i). Long‐term potentiation (LTP) is believed to the cellular basis of learning and memory. The LTP of field excitatory postsynaptic potentials (fEPSP) in the hippocampal CA1 region was decreased in TRPC1‐overexpressed hippocampi compared with controls (Figure 3j,k). Golgi staining revealed that the dendritic spines in the hippocampal CA3 were significantly diminished upon TRPC1 overexpression as compared with the controls (Figure 3l,m). These in vivo data demonstrate that overexpression of TRPC1 induces synaptic impairments and cognitive deficits.

### 2.4 Inhibiting TRPC1 attenuates hTau‐induced SOCE/ER stress and kinases/phosphatase dysregulation with tau dephosphorylation in vitro

To further verify the role of TRPC1 in hTau‐induced dysregulations, we used a pan TRPC inhibitor (named SKF96365) in cultured primary neuron. After transfecting lenti‐hTau for 7 days, the neurons were treated with SKF96365 (15 and 30 μM) or the vehicle (0.1% DMSO) for 90 min. We observed that SKF96365 treatment efficiently attenuated hTau‐induced ER stress and restored the activities of AKT/GSK3β, CaMKII, and PP2A, indicated by the restored levels of GRP78, p‐PERK (Thr980), p‐AKT (Ser473), p‐GSK3β (Ser9), p‐CaMKII (Thr286), and PP2A‐C (Figure S1a–d). Accordingly, the increased total tau and the hyperphosphorylated tau at p‐T181, AT8, p‐S396 epitopes were also restored by SKF96365 (Figure S1e,f). These data demonstrate that inhibiting TRPC1 could effectively attenuate ER stress and restore kinases/phosphatase activity with restoration of tau phosphorylation.

**FIGURE 4:**
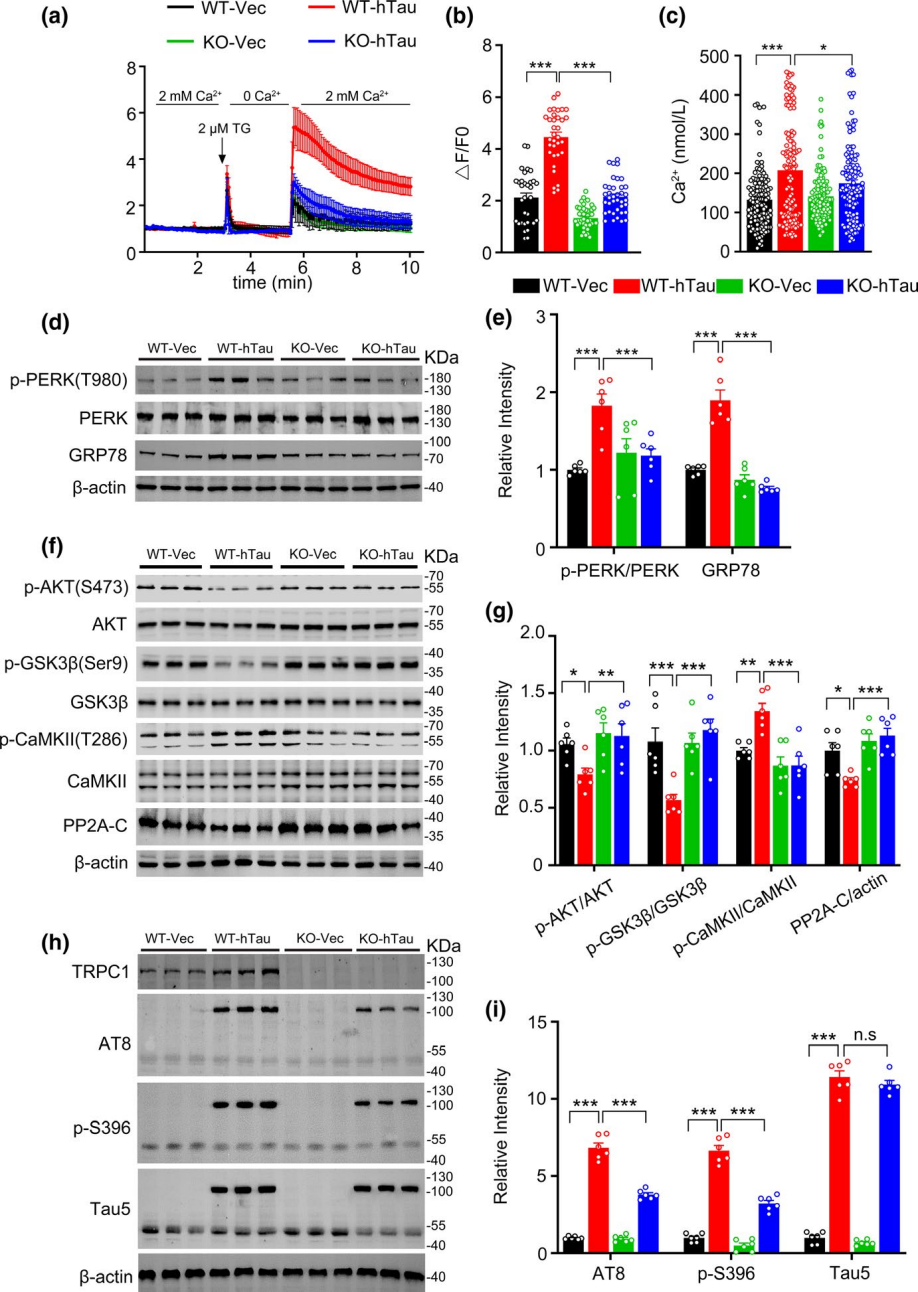
TRPC1 knockout attenuates hTau‐induced SOCE hyperactivation and intracellular Ca^2+^ overloading with attenuated ER stress/kinases/phosphatase dysregulation and decreased tau burden. (a, b) TRPC1 knockout attenuates hTau‐induced SOCE hyperactivation. The primary hippocampal neurons dissected from the hippocampi of TRPC1‐wild type (WT) and TRPC1^−/−^ (KO) mice were cultured for 5 days in vitro (5 *div*), and then infected with lenti‐syn‐hTau‐mCherry or the empty vector for another 7 days, and then, the neurons were loaded with Fluo3‐AM for measurement of intracellular [Ca^2+^]_i_. The time course of intracellular Ca^2+^ signals were measured by following the store‐depletion protocol and the amplitude of Ca^2+^ influx was presented. *N* = 32–42 neurons per group, one‐way ANOVA, Dunnett's post hoc analysis. (c) TRPC1 knockout attenuates hTau‐induced intracellular Ca^2+^ overload measured by confocal Ca^2+^ imaging. *N* = 126–133 neurons per group, one‐way ANOVA, Dunnett's post hoc analysis. (d, e) TRPC1 knockout attenuates ER stress in vivo. AAV‐syn‐TRPC1‐eGFP or the empty vector was infused into the hippocampal CA3 of 2‐month‐old TRPC1WT and TRPC1^−/−^ (KO) mice for one month, then the extracts of hippocampal CA3 subset was used for Western blotting. The phosphorylated level was normalized to the total level of each protein. *N* = 6 per group, one‐way ANOVA, Dunnett's post hoc analysis. (f, g) TRPC1 knockout attenuates hTau‐induced dysregulation of AKT/GSK‐3β/CaMKII/PP2A in hippocampal CA3 extracts of mice. *N* = 6 per group, two‐way ANOVA, Dunnett's post hoc analysis. (h, i) TRPC1 knockout arrests hTau‐induced tau hyperphosphorylation at AT8, p‐S262, p‐S396 with minor effect on total tau probed by Tau5. β‐actin was used as a loading control. *N* = 6 per group, one‐way ANOVA, Dunnett's post hoc analysis. Data were presented as mean ± SD for (a) and mean ± SEM for (b–i), *p < 0.05, **p < 0.01, ***p < 0.001

TRPC1 is a Ca^2+^ permeable nonselective cation channel that can be activated by ER store depletion. Ca^2+^ entry through TRPC1 leads to a sustained increase in intracellular Ca^2+^ concentrations (Birnbaumer, 2009). Overexpressing hTau could increase the level of intracellular basal [Ca^2+^] in hippocampal neurons (Yin, Wang, et al., 2016). To investigate whether TRPC1 knockout could rescue hTau‐induced SOCE activation and the intracellular Ca^2+^ overload, we cultured the primary hippocampal neurons dissected at embryonic days 17 to 19 from TRPC1 knockout or the wild‐type mice. The neurons at 5 *div* were transfected with lenti‐syn‐hTau‐mCherry or the lenti‐syn‐mCherry. After 7 days, the amplitude of SOCE and intracellular [Ca^2+^]_i_ were measured. TRPC1 knockout abolished the hTau‐induced SOCE enhancement (Figure 4a,b) with a simultaneous reduction of the intracellular [Ca^2+^]_i_ compared with the wild‐type neurons (Figure 4c). These data suggest that TRPC1 is responsible for hTau‐induced SOCE strengthening and intracellular Ca^2+^ overload.

### 2.5 TRPC1 knockout ameliorates hTau‐induced ER/kinases/phosphatase/tau pathologies with improved synaptic and cognitive functions

**FIGURE 5:**
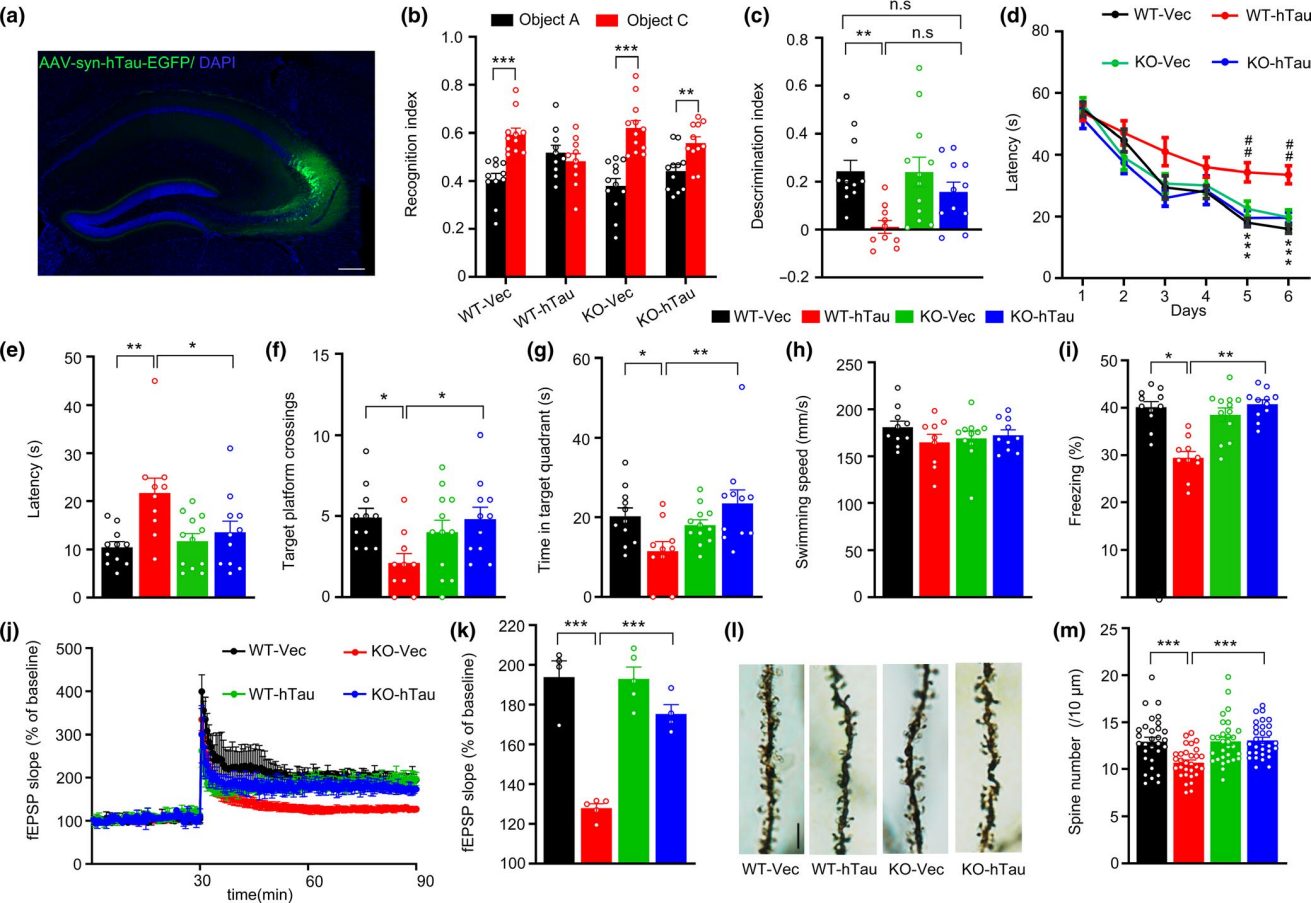
TRPC1 knockout improves hTau‐induced memory deficits and synaptic dysfunction. (a) The representative image showing expression of hTau in 2‐month‐old mice. AAV‐syn‐hTau‐eGFP or the empty vector was stereotaxically infused into the hippocampal CA3 of 2‐month‐old TRPC1‐WT or TRPC1^−/−^ (KO) mice. After one month, the expression of AAV‐syn‐hTau‐eGFP was confirmed by confocal microscopy, scale bar, 100 μm. (b, c) TRPC1 knockout rescues hTau‐induced memory deficit measured by novel object recognition (NOR) test, shown by the reversed recognition index (b) and the discrimination index (c) recorded at 24 hr after training. *N* = 10–12 mice per group, one‐way ANOVA, Dunnett's post hoc analysis. (d–h) TRPC1 knockout attenuates hTau‐induced spatial learning and memory deficits shown by the decreased latency to find the hidden platform during 6 days training on MWM (d), and the decreased latency to find the platform site (e), the increased target platform crossing (f) and the duration in the target zone measured at day 8 after removed the platform (g). The swimming velocity was not changed (h). *N* = 10–12 mice per group, one‐way ANOVA, Dunnett's post hoc analysis. (i) TRPC1 knockout attenuates hTau‐induced contextual memory impairment shown by attenuated total freezing time during 3 min test. *N* = 10–12 mice per group, one‐way ANOVA, Dunnett's post hoc analysis. (j, k) TRPC1 knockout rescues hTau‐induced inhibition of LTP induction shown by the restored fEPSP slope induced by applying 3 trains of high‐frequency stimulation (HFS). *N* = 4–5 mice per group, one‐way ANOVA, Dunnett's post hoc analysis. (l, m) TRPC1 knockout rescues hTau‐induced spine impairments measured by Golgi staining. *N* = 30 neurons from 3 mice per group, one‐way ANOVA, Dunnett's post hoc analysis. The data were expressed as mean ± SD for (j) and mean ± SEM for (b–i) and (k, m). *p < 0.05, **p < 0.01, ***p < 0.001

To confirm the role of TRPC1 in mediating and aggravating tau pathologies *in vivo*, we injected stereotaxically AAV‐syn‐hTau‐eGFP or AAV‐syn‐eGFP viruses into the dorsal hippocampal CA3 of 2‐month‐old wild‐type (TRPC1WT) and TRPC1 knockout (TRPC1KO) mice, respectively. One month later, the expression pattern of AAV‐syn‐TRPC1‐eGFP was confirmed by fluorescence imaging (Figure 5a). Consistent with the in vitro results, TRPC1 knockout attenuated hTau‐induced dysregulation of GRP78, p‐PERK (Thr980), p‐AKT (Ser473), p‐GSK3β (Ser9), p‐CaMKII (Thr286), and PP2A‐C (Figure 4d–g). Simultaneously, the phosphorylation level of tau at AT8 and p‐S396 epitopes in hTau‐overexpressing TRPC1^−/−^ (TRPC1KO‐hTau) mice was also decreased compared to TRPC1WT‐hTau mice (Figure 4h,i). These in vivo data further confirm the critical role of TRPC1 in mediating and aggravating hTau pathologies.

A series of behavioral tests were performed to validate cognitive function of the mice. During NOR test, the recognition index of the four group mice was comparable in the training trial. In the test trail, 24 hr after the training, the TRPC1WT/hTau mice showed decreased recognition index (Figure 5b) and discrimination index (Figure 5c) to the novel object, while the hTau‐induced deficit was attenuated by TRPC1 knockout. During MWM test, overexpressing hTau in TRPC1WT mice induced significant learning deficit with the highest latency at days 3, 5, and 6, whereas TRPC1 knockout improved learning ability of the mice (Figure 5d). Spatial memory was evaluated at day 8 after removed the platform. We observed that TRPC1 knockout could efficiently rescue the hTau‐induced spatial memory deficit shown by decreased latency to reach the platform site (Figure 5e), more frequent crossings (Figure 5f) and increased time spend in the target quadrant (Figure 5g). The swimming speed was similar among the four groups (Figure 5h). Fear conditioning test also showed that TRPC1 knockout increased freezing time compared with TRPC1WT/hTau mice (Figure 5i). Notably, TRPC1KO mice expressing AAV‐syn‐eGFP empty vector did not show significant difference in learning and memory abilities compared with WT mice (Figure 5b–i). These data demonstrate that TRPC1 knockout attenuates hTau‐induced cognitive deficits in mice.

By electrophysiological recordings, we observed a significant LTP reduction in TRPC1WT/hTau mice compared with the control group (TRPC1WT/empty vector), and this inhibition was attenuated by TRPC1 knockout (Figure 5j,k). Analysis of dendritic spines from hippocampal slices also confirmed that TRPC1 knockout restored hTau‐induced impairments on spine formation (Figure 5l,m). These data demonstrate that TRPC1 plays a critical role in mediating hTau‐induced synaptic impairments.

### 2.6 Overexpressing hTau upregulates TRPC1 through activating C/EBPβ

**FIGURE 6:**
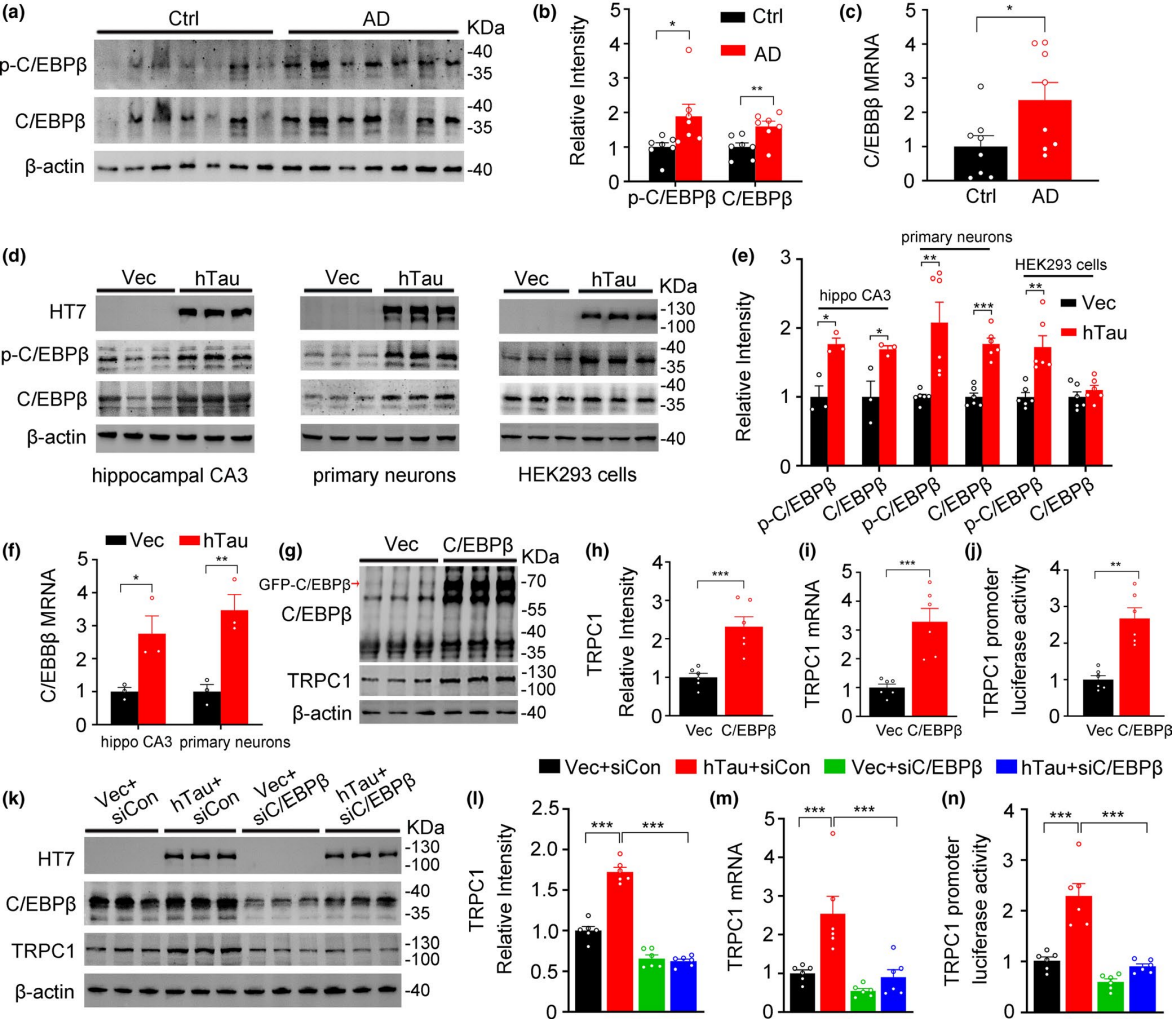
Overexpressing hTau increases TRPC1 through activating C/EBPβ. (a–c) Total and the activated C/EBPβ (phosphorylated at Thr235/188 sites) protein and the mRNA levels were increased in the hippocampal extracts of the AD brains measured by Western blotting and qPCR. *N* = 7–8 for each group, unpaired Student's *t*‐test. (d–f) Overexpressing hTau upregulates C/EBPβ shown by the increased total and p‐C/EBPβ (Thr235/188) and mRNA levels in the extracts of hippocampal CA3, the primary cultured hippocampal neurons (*div* 5 + 7), and HEK293 cells transfected with hTau or vector plasmids for 48 hr. β‐actin was used as a loading control. *N* = 3 for hippocampal CA3; *N* = 6 for primary neurons and HEK293 cells, unpaired Student's *t*‐test. (g–j) Overexpressing C/EBPβ increases TRPC1 transcription and translation. HEK293 cells were transfected with eGFP‐C/EBPβ or the empty vector for 48 hr, and then the levels of TRPC1 protein, mRNA and promotor luciferase activity were measured. *N* = 6 for each group, unpaired Student's *t*‐test. (k–n) Downregulating C/EBPβ rescues hTau‐induced elevation of TRPC1 transcription and translation. HEK293 cells were co‐transfected with hTau or the empty vector and siC/EBPβ or siCon for 48 hr, and then the levels of TRPC1 protein, mRNA and promotor luciferase activity were measured. *N* = 6 per group, one‐way ANOVA, Dunnett's post hoc analysis. Data were expressed as mean ± SEM. *p < 0.05, **p < 0.01, ***p < 0.001

Given that TRPC1 mRNA is increased in hTau‐overexpressing neurons and the AD brain (Figure 1c,f), the involvement of TRPC1 transcription is suggested. To verify this, we screened for potential transcription factors that mediate TRPC1 mRNA expression by using “AliBaba2.1,” which predicts the transcription factors by constructing matrices on the fly from TRANSFAC 4.0 sites. Among the dozens of putative transcription factors from website predicting, we found that C/EBPβ (CCAAT/enhancer binding protein β) maybe a well‐conserved candidate since C/EBPβ is an aging‐related transcription factor indicated in the GenAge Database, abundantly expressed in the brain and can be activated by AD‐risk factors (Chai et al., 2017). To confirm whether C/EBPβ is activated in AD, we detected the levels of phosphorylated and total C/EBPβ in hippocampus of human AD brain. Both p‐C/EBPβ (Thr235/188, activated sites) and total C/EBPβ and as well as the mRNA level of C/EBPβ were increased in the human AD brain (Figure 6a–c). We also detected p‐C/EBPβ and total C/EBPβ in mouse hippocampal CA3 extracts (samples were made as the same process mentioned in Figure 4), the primary hippocampal neuron lysates (samples were made as the same process described in Figure 1) and HEK293 cell lysates (transfected with p‐IRES‐eGFP‐Tau40 or the vector p‐IRES‐eGFP plasmid for 48 h), and activation of C/EBPβ by hTau was detected both in vitro and in vivo (Figure 6d,e). The increased C/EBPβ mRNA was also shown in cultured neurons by overexpressing hTau (Figure 6f). These data confirm that overexpressing hTau upregulates C/EBPβ, which could be upstream of the increased TRPC1 by hTau overexpression (Tables 1 and 2).

**TABLE 1:** Primary antibodies used in the study

Antibody	Specificity/Immunogen	Host	Dilution	Catalogue number
HT7	Total/human aa 159‐163	M	1:1000 for WB	Thermo Fisher scientific, MN1000
Tau 5	Total/full length purified Cow Tau	M	1:1000 for WB	Abcam, ab80579
AT8	Partially purified human PHF‐tau	M	1:1000 for WB 1:100 for IF	Thermo Fisher scientific, MN1020
p‐S262	Phosphorylated around Ser262 (I‐G‐S(p)‐T‐E)/human	R	1:1000 for WB	Sabbiotech, 11111
p‐S396	Phosphorylated around Ser262 (Y‐K‐S(p)‐P‐V)/human	R	1:1000 for WB 1:100 for IF	Sabbiotech, 11102
p‐T181	Phosphorylated around aa 179‐183/human	R	1:1000 for WB	Sabbiotech, 21096
TRPC1	Total/human synthetic peptide	R	1:1000 for WB	Abcam, ab51255
TRPC1	Total/human aa 557‐571	R	1:1000 for WB 1:100 for IF&IHC	Sigma‐Aldrich, T8276
ORAI1	Total/human 18 aa peptide from near the amino terminus	R	1:1000 for WB	Abcam, ab59330
AKT	Total/mouse synthetic peptide corresponding to carboxyl terminal sequence	R	1:1000 for WB	Cell signaling Technology, 9272
p‐AKT(Ser473)	Phosphorylated around Ser473 /mouse	R	1:1000 for WB	Cell signaling Technology, 4058
p‐AKT(Thr308)	Phosphorylated around Thr308 /mouse	R	1:1000 for WB	Cell signaling Technology, 9275
GSK3β	Total/human synthetic peptide corresponding to carboxyl terminal sequence	R	1:1000 for WB	Cell signaling Technology, 12456
p‐GSK3β(Ser9)	Phosphorylated around Ser9/human	M	1:1000 for WB	Cell signaling Technology, 14630
PP2A‐C	Total/human synthetic peptide corresponding to carboxyl terminal sequence	M	1:1000 for WB	Cell signaling Technology, 2038
PP2A‐B	Total/human synthetic peptide corresponding to carboxyl terminal sequence	R	1:1000 for WB	Cell signaling Technology, 4953
CaMKII	Total/human synthetic peptide corresponding to amino terminal sequence	R	1:1000 for WB	Cell signaling Technology, 3362
p‐CaMKII(Thr286)	Phosphorylated around Thr287/human	R	1:1000 for WB	Cell signaling Technology, 12716
PERK	Total/human aa 850 to the C‐terminus	R	1:500 for WB	Abcam, ab229912
p‐PERK(Thr980)	Phosphorylated around Thr980/mouse	R	1:500 for WB	Cell signaling Technology, 3179
IRE1	Total/human 16 aa from near the carboxyl terminus	R	1:1000 for WB	Abcam, ab37073
p‐IRE1(Ser724)	Phosphorylated at Ser724/human	R	1:1000 for WB	Abcam, ab48187
ATF6	Total/human 12 aa from near the carboxyl terminus	R	1:1000 for WB	Abcam, ab62576
GRP78	Total/mouse aa 600 to the C‐terminus	R	1:1000 for WB	Abcam, ab21685
C/EBPβ	Total/rat aa 250‐350	R	1:1000 for WB	Abcam, ab32358
p‐ C/EBPβ	Phosphorylated around the phosphorylation site of Thr235/188	R	1:500 for WB	Abcam, ab52194
β‐actin	Total/aa 1‐14	M	1:2000 for WB	Abcam, ab6272
GAPDH	Total/rabbit muscle GAPDH	M	1:1000 for WB	Abcam, ab8245

WB: Western blotting; IP: immunoprecipitation; IF: immunofluorescence; IHC: immunohistochemistry; aa: amino acid; M: mouse; R: rabbit.

**TABLE 2:** Information for human brain samples

Case number	Gender	Age	Neuropathological Diagnosis	Postmortem Interval (h)
PTB078	F	86	AD	6.33
PTB079	F	80	AD	18
PTB083	F	100	AD	3
PTB108	M	72	AD	
PTB114	F	80	AD	13
PTB129	M	83	AD	4.5
PTB139	M	82	AD	38
PTB142	M	97	AD	26.5
PTB041	M	80	Control	7.5
PTB054	F	84	Control	20
PTB088	M	79	Control	29
PTB140	M	83	Control	38
PTB144	F	96	Control	32
PTB158	M	86	Control	5
PTB186	F	87	Control	12.5
PTB187	F	81	Control	7

Abbreviations: AD, Alzheimer's disease; F, female; M, male.

To confirm the role of C/EBPβ in upregulating TRPC1, we overexpressed eGFP‐C/EBPβ and measured TRPC1 level in HEK293 cells after 48 hr. The increased protein and mRNA levels (Figure 6g–i) and luciferase activity of TRPC1 promotor (Figure 6j) were detected after C/EBPβ overexpression. Moreover, downregulating C/EBPβ attenuated hTau‐induced increasement of TRPC1 protein and mRNA levels, and as well as the luciferase activity of TRPC1 promoter (Figure 6k–n). These data indicate that overexpressing hTau increases TRPC1 transcription through activating C/EBPβ.

## 3 DISCUSSION

Abnormally hyperphosphorylated wild‐type tau is the major protein component of neurofibril tangles in the brains of sporadic AD patients. Multiple phosphorylation sites of tau have been identified in the AD brains and some of them are correlated with severity of neuronal cytopathy (Augustinack, Schneider, Mandelkow, & Hyman, 2002). Hyperphosphorylation induces mis‐localization of tau from axons to soma and dendritic compartment, alters tau degradation and truncation by proteases, and enhances tau accumulation, which together leads to synaptic dysfunction and memory deficits (Wang & Mandelkow, 2016). Therefore, timely blocking tau hyperphosphorylation could be promising to arrest neurodegeneration. However, the molecular mechanisms leading to a persistent and aggravated tau hyperphosphorylation remain unclear. In the present study, we demonstrate that increasing intracellular tau can upregulate C/EBPβ‐TRPC1‐SOCE signaling and thus induces ER stress and an imbalanced protein kinases and phosphatase, which can be the direct cause for a persistent and aggravated tau hyperphosphorylation. Downregulating TRPC1 efficiently rescues hTau‐induced SOCE and intracellular Ca^2+^ overload with improved tau phosphorylating system and synaptic functions, and downregulating C/EBPβ attenuates hTau‐induced upregulation of TRPC1. These findings reveal that increasing tau can serve as an upstream factor to activate C/EBPβ‐TRPC1‐SOCE‐ER‐kinase/phosphatase axis, leading to a persistent and aggravated tau hyperphosphorylation and eventually neurodegeneration, while targeting TRPC1 or C/EBPβ could be promising to arrest the aggravating tauopathies during AD progression.

Accumulation of the hyperphosphorylated tau induces synapse and memory deficits in mice (Li et al., 2019; Ye et al., 2019; Yin, Gao, et al., 2016), but the molecular mechanisms are not fully understood. By neuron‐specific overexpression of hTau in primary hippocampal neurons, we found a hyperactivated SOCE signaling, activated ER stress/AKT/GSK3β pathway, increased Ca^2+^/CaMKII, and decreased PP2A‐C expression. Among these kinases and phosphatase, CaMKII is activated by the binding of Ca^2+^/CaM followed by autophosphorylation at Thr286 (Yoshimura, Ichinose, & Yamauchi, 2003). CaMKII phosphorylates tau at Ser262/356 which co‐colocalizes with neurofibrillary tangles in the AD brains (Xiao, Perry, Troncoso, & Monteiro, 1996). The intracellular accumulation of hTau increased intracellular basal [Ca^2+^]_i_ and hyperactivation of SOCE (Yin, Wang, et al., 2016). Thus, hTau may upregulate CaMKII phosphorylation through SOCE. We also observed activation of GSK‐3β, one of the most recognized tau kinases, by hTau. GSK‐3β is activated in the brains of AD patients and the mouse models (Ali & Kim, 2015; Hooper, Killick, & Lovestone, 2008), overexpression of GSK‐3β in adult mouse brain induces tau hyperphosphorylation and consequently neurodegeneration (Lucas et al., 2001). Phosphorylation of GSK‐3β at Ser‐9 by AKT inhibits its activity; thus, the ER stress‐induced inhibition of AKT results in GSK‐3β activation by a reduced Ser‐9 phosphorylation. As GSK‐3β activity is also regulated by intracellular [Ca^2+^]_i_ (Yuan et al., 2015), the hTau‐induced increase of SOCE at least contributes the activated GSK‐3β. We also noticed that PP2A‐C was reduced by overexpressing hTau without changing PP2A‐B. PP2A inactivation is regarded as the most important molecular event that causes abnormal tau hyperphosphorylation in the AD brains (Iqbal, Liu, & Gong, 2016). PP2A‐C expression can be inhibited by increased intracellular [Ca^2+^]_i_ through degradation (Chiou et al., 2019) and activated ER stress (Tay et al., 2012). Therefore, the reduction of PP2A‐C in hTau‐overexpressing neurons may be caused by activated ER stress and elevated intraneuronal [Ca^2+^]_i_. Weak positive signals of AT8, p‐S262, and p‐S396 could be detected in the normal brains; however, these phosphorylation sites were remarkably increased in the AD brains, which was correlated with the severity of neurodegeneration. Our results showed that overexpressing TRPC1 increased tau phosphorylation at these sites and elicited cognitive deficits, while pharmacological inhibiting or knockout TRPC1 attenuated these tau phosphorylation sites with improved synaptic and cognitive functions. Thus, the increasement of AT8, p‐S262, and p‐S396 levels may play a crucial role in mediating the toxic effects of TRPC1 upregulation. Additionally, the decreased PP2A may attenuate GSK3β dephosphorylation at Ser9 (Wang et al., 2015); however, we observed a reduced GSK3β phosphorylation by TRPC1 activation. As TRPC1 activation also induced AKT dephosphorylation which may lead to a reduced GSK3β phosphorylation, we speculate that the SOCE hyperactivation‐induced AKT inhibition may overwhelm the effect of PP2A inhibition on GSK3β phosphorylation. To conclude, increasing intracellular hTau induces dysregulation of AKT/GSK‐3β, CaMKII, PP2A via increasing TRPC1‐SOCE signaling, which in turn causes a persistent tau hyperphosphorylation, forming a vicious circle.

We observed elevated TRPC1 with an enhanced SOCE in hTau‐overexpressing cultured neurons and the human AD hippocampus. TRPC1, a Ca^2+^‐permeable nonselective cation channel, is a component of SOCE and can be activated in response to Ca^2+^ store depletion (Birnbaumer, 2009). Both brain cortex and the hippocampus are highly susceptible to calcium disorder and vulnerable to tau aggregation in the AD progression. We noticed that TRPC1 level was much lower in the hippocampus than the cortex in the normal control brains, which might provide sufficient biological foundation for TRPC1 elevation in the hippocampus. However, the cortex level of TRPC1 in both AD and control brains was as high as its level in the AD hippocampus, suggesting a ceiling effect in the cortex. Our result was also in accordance with previous study which revealed a limited effected level of TRPC1 protein in AD cortical lysates (Sun et al., 2014). We found that knockout of TRPC1 eliminated hTau‐induced SOCE hyperactivation and intraneuronal Ca^2+^ overloading. Thus, we speculate that the elevated TRPC1 can boost SOCE. SOCE plays a significant role in Ca^2+^ dysregulation. The currently reported role of TRPC1 and SOCE in synapse stabilization and neurodegeneration is not always consistent. Some studies show that the impaired SOCE causes destabilization of mature spines through STIM2‐nSOC‐CaMKII pathway in both PS1‐M146V‐KI and APP‐KI mouse models of AD (Sun et al., 2014; Zhang et al., 2015). Expressing PS1 delta E9 mutant induces STIM1‐driven store‐operated Ca^2+^ channel hyperactivation in hippocampal neurons (Ryazantseva et al., 2018). Enhanced SOCE leads to synaptic loss while inhibiting TRPC1‐dependent SOCE improves synaptic stability and motor performance in a mouse model of HD (Wu et al., 2016) (Wu, Ryskamp, Birnbaumer, & Bezprozvanny, 2018). We observed that TRPC1 knockout rescues synaptic impairment and memory deficit in hTau‐overexpressing mice. Treatment with a pharmacological TRPC1 inhibitor SKF96365 or knockout of TRPC1 also blocked hTau‐induced activation of ER stress/AKT/GSK3β and CaMKII and suppression of PP2A, which in turn reduced tau hyperphosphorylation. We also noticed that TRPC1 inhibitor (SKF96365) did not affect basal activity of TRPC1 and application of the inhibitor at 15 or 30 μM showed similar efficiency in attenuating the hTau‐induced kinases‐phosphatase balance and tau phosphorylation level. We speculate that 15 μM SKF96365 may have reached the plateau of its attenuating effects, thus increasing the concentration to 30 μM could not further decrease tau phosphorylation. In future studies, lower concentrations of SKF96365 may be tested in different experimental systems. Importantly, we also observed that TRPC1‐null mice showed comparable learning and memory with wild‐type mice, implicating the advantage of TRPC1 as a good potential therapeutic target. Therefore, TRPC1 may serve as a promising therapeutic target for tauopathies.

Intriguingly, we observed that overexpressing TRPC1 in cultured hippocampal neurons only selectively activated PERK pathway without affecting the other ER stress pathways, although the ER stress chaperone GRP78 was elevated. ER stress is activated by the coordinated activation of three ER transmembrane stress sensors: IRE1α, PERK, and ATF6 (Grootjans, Kaser, Kaufman, & Blumberg, 2016). Under homeostatic conditions, the luminal domains of these ER stress sensors are retained in an inactive state through association with GRP78. However, when misfolded proteins accumulate in the ER lumen, GRP78 dissociates from ER stress sensors and thereby release the stress sensors to permit downstream signaling (Grootjans et al., 2016). Studies have shown that PERK is not only activated by the accumulation of unfolded proteins in ER under stress conditions, elevation of cytosolic Ca^2+^ also activates PERK (Wang et al., 2013). TRPC1 overexpression results in increased cytosolic Ca^2+^, which may be another interpretation for selective PERK activation. PERK was identified as a genetic risk factor in several tauopathies (Yuan et al., 2018), inhibiting PERK prevents tau‐mediated neurodegeneration in rTg4510 mice (Radford, Moreno, Verity, Halliday, & Mallucci, 2015). We found that both pharmacological inhibition and genetic knockout of TRPC1 blocked hTau‐induced GRP78 elevation and PERK activation.

To explore the mechanism underlying hTau‐induced TRPC1 mRNA elevation, we screened for the potential transcription factors that mediate TRPC1 mRNA expression. We found that C/EBPβ, a well‐conserved element in the proximal promotor region of TRPC1, is activated in hTau‐overexpressing cells, in the hippocampi of hTau‐overexpressing mice and the hippocampi of human AD brain. As a member of C/EBP family, C/EBPβ is implicated in regulating inflammatory genes in concert with nuclear factor κB (NFκB) and it is activated in the aging and AD brains (Chai et al., 2017). We observed that overexpressing CEBP/β increased TRPC1 promotor luciferase activity, mRNA transcription and protein expression, while downregulating C/EBPβ attenuated hTau‐induced elevation of TRPC1 transcriptional and translational activity. In consistent with this finding, recent studies also show that overexpressing CEBP/β in young AD mice accelerates earlier onset of AD‐like pathologies and knockout of CEBP/β noticeably reduces neurofibrillary tangles in 3xTg mice (Wang, Liu, Chen, & Ye, 2018; Wang, Gong, et al., 2018).

Taken together, our current study reveals that increasing intracellular tau can upregulate C/EBPβ/TRPC1/SOCE to induce ER stress and dysregulation of protein kinases/phosphatase, which in turn aggravates tau hyperphosphorylation and the toxicities, while inhibiting TRPC1 can efficiently block this vicious cycle.

## 4 MATERIALS AND METHODS

Please see the Appendix S1.

## CONFLICT OF INTEREST

The authors declare no competing financial interests.

## AUTHOR CONTRIBUTIONS

Author contribution: Conceptualization, J.W.Y. and J.Z.W.; Investigation, J.W.Y., Y.L.Y., H.Q.Z., Y. Y., H.L.W., L W., D.G., M.Z.L., Y.C.L. and K.D.; Writing manuscript, J.W.Y. and J.Z.W.; Funding Acquisition, Y.L.Y. and J.Z.W.; Resources, H.Q.Z. and J.Z.W.; Supervision, H.Q.Z., J.L. and J.Z.W.

## Supporting information

Fig S1

AppendixS1

## Data Availability

The data that support the findings of this study are available on request from the corresponding author. The data are not publicly available due to privacy or ethical restrictions.
