# Supplementary material for: Tau‐induced upregulation of C/EBPβ‐TRPC1‐SOCE signaling aggravates tauopathies: A vicious cycle in Alzheimer neurodegeneration
Source: Aging Cell. 2020 Aug 20;19(9):e13209. doi: 10.1111/acel.13209 (PMC7511862; doi:10.1111/acel.13209)
Supplement: Supplementary file 1 — Fig S1 [file ACEL-19-e13209-s001.docx]

**Supplementary Figure 1**


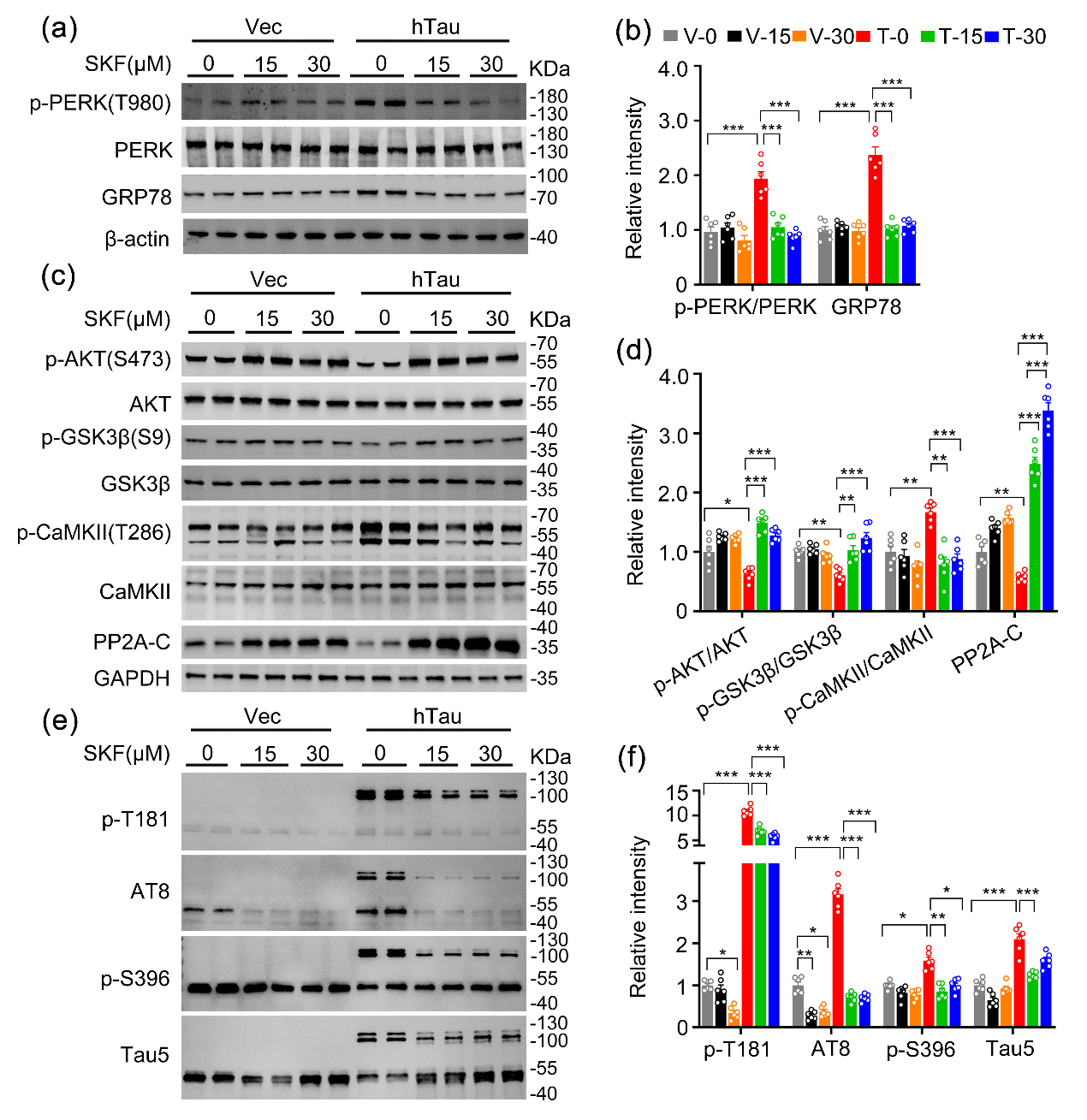


**S-Figure 1. Inhibiting TRPC1 attenuates hTau-induced ER stress and protein kinases/phosphatase imbalance with reduced tau phosphorylation.**

(a, b) Inhibiting TRPC1 by SKF96365 attenuates hTau-induced ER stress. The hippocampal neurons (5 *div*) were infected with lenti-syn-hTau-mCherry or lenti-syn-mCherry for 7 days, then the neurons were treated with 15 μM or 30 μM SKF96365 or the vehicle for 1.5 h. The levels of p-PERK (Thr980), PERK, GRP78 were detected by Western blotting. N = 6 per group, one-way ANOVA, Tukey’s post hoc analysis.

(c, d) Inhibiting TRPC1 attenuates hTau-induced imbalance of kinases and PP2A measured by Western blotting. N = 6 per group, one-way ANOVA, Tukey’s post hoc analysis.

(e, f) Inhibiting TRPC1 decreases hTau-induced hyperphosphorylation of tau at AT8, p-S262, p-S396 epitopes with reduction of total tau probed by Tau5. N = 6 for each group, one-way ANOVA, Tukey’s post hoc analysis.

Data were expressed as mean ± SEM, **p* < 0.05, ***p* < 0.01, ****p* < 0.001.
