# Supplementary material for: Tau‐induced upregulation of C/EBPβ‐TRPC1‐SOCE signaling aggravates tauopathies: A vicious cycle in Alzheimer neurodegeneration
Source: Aging Cell. 2020 Aug 20;19(9):e13209. doi: 10.1111/acel.13209 (PMC7511862; doi:10.1111/acel.13209)
Supplement: Supplementary file 2 — AppendixS1 [file ACEL-19-e13209-s002.docx]

**SUPPORTING INFORMATION**

**MATERIALS AND METHODS**

**1.1 | Plasmids, Viruses, Reagents, and Antibodies**

The pEGFP-hTau plasmid containing the wild-type full-length 2N4R human tau (hTau) was a kind gift from Dr. Fei Liu (Jiangsu key Laboratory of neurodegeneration, Nantong University, China). The lenti-syn-TRPC1-eGFP, lenti-syn-eGFP, the eGFP-unfused lenti-syn-TRPC1-T2A-eGFP, and lenti-syn-T2A-eGFP, AAV-syn-TRPC1-eGFP, AAV-syn-eGFP, lenti-syn-hTau-mCherry, lenti-syn-mCherry, AAV-syn-hTau-eGFP were constructed and packaged by Obio Technology (Shanghai, China). The pEGFP-CEBP/β plasmid and TRPC1 luciferase reporter plasmid (pGLC basic luciferase vector containing the -2000 to +100 region) were constructed by TsingKe Biotechnology (Beijing, China). The CEBP/β siRNA was purchased from Santa Cruz Biotechnology (sc-29229). AAV2/8 was used in animal experiments. SKF96365 (a TRPC channel inhibitor) was from Selleck (USA). SYBR Premix Ex Taq^TM^ II (TliRNaseH Plus) (used for real-time qPCR) was from Takara (Japan). All other reagents were from Sigma-Aldrich. All the primary antibodies used in the study are listed in Table 1.

**1.2 | Cell Culture and Transfection**

HEK293 cells were cultured with DMEM-high glucose medium supplemented with 10% fetal bovine serum (FBS), 100U/mL penicillin, and 0.1mg/mL streptomycin (all from Hyclone) at 37℃ in the presence of 5% CO_2_. HEK293 cells transfection was performed with lipoFactMax^TM^ (ABP biosciences, Beltsville, USA).

Primary hippocampal neuron culture was performed as described before (Ye *et al.* 2019), neurons were isolated from 17d- to 19d-embryonic Sprague Dawley rats or TRPC1KO and wild-type mice. Hippocampus were isolated and neurons were planted with the F-12 medium containing 10% FBS on a coverslip in a 12-well plate with 20, 000 cells per well or on a 6-well plate with 100, 000 cells per well. Four hours after planting, medium was replaced with 1.5 ml of fresh maintenance medium containing 97% neurobasal medium, 2% B27, and 1% GlutaMAX. At 5 *div*, neurons were infected with lentivirus with a multiplicity of infection (MOI) of 10. Half of the maintenance medium was changed every 3 days.

**1.3 | Luciferase Reporter Assay**

HEK293 cells were planted in 24-well plates one day prior transfection and transfected with luciferase reporter plasmid together with pRL-TK Renilla luciferase plasmid (Progema), as well as eGFP-hTau or siCEBP/β plasmids. Forty-eight hours after transfection, the cells were harvested and lysed with 100 μL Passive Lysis Buffer. Luciferase activity were analyzed using a Lumat LB9507 luminometer (Berthold) and the Dual-luciferase Reporter Assay Systerm (Promega) according to the manufacturer’s protocol (Progema). Relative light units of TRPC1 luciferase were normalized to Renilla luciferase light units to control for transfection efficiency.

**1.4 | Ca^2+^ Imaging**

Ca^2+^ imaging was performed as described before (Yin *et al.* 2016b). Primary hippocampal neurons were plated on the coverslip (diameter = 30 mm) coated with 100 μg/ml poly-D-lysine and cultured for 7 days and then infected with lenti-syn-hTau-mCherry or lenti-syn-mCherry for another 5 days. After incubation with the calcium dye Fluo3-AM (5 μM, Dojindo, Japan) at 37°C for 30 min in Krebs-HEPES buffer containing (in mM): 135 NaCl, 6KCl, 2 CaCl_2_, 1.2 MgCl_2_, 10 D-glucose, and 10 HEPES at pH 7.4, cells were washed for 3 times and incubated with Krebs-HEPES buffer for de-esterification at 37°C for 20 min. Then, the coverslip was mounted into a perfusion chamber and fluorescence signals were recorded by the time-series scan imaging with a confocal microscopy (Zeiss LSM 780, 40X, 1.3 NA objectives). Two thirds of the cell soma were set as the region of interest (ROI) for imaging scan. The real-time recorded signals (F) were normalized to the mean baseline recorded at the first 3 min (F_0_), which is expressed as F/ F_0_. To determine the [Ca^2+^]_i_, high Ca^2+^ solution (5 mM Ca^2+^) and Ca^2+^-free/EGTA solution containing Ca^2+^ ionophore ionomycin (10 μM) were perfused, respectively. The fluorescence was acquired as F_max_ and F_min_. For the recorded signals F, [Ca^2+^]_i_ = [(F- F_min_)/ (F- F_max_)] x K_d_, K_d_ = 400 nM.

**1.5 | Western Blotting**

The cells were collected and lysed with RIPA buffer on ice for 10 min and then centrifuged at 12, 000 ×g for 10 min at 4°C. The mice brain subset hippocampal CA3 were separated with vibrating microtome (Leica, VT1000S, Germany) on ice-cold PBS according to the mouse brain atlas, and then, the CA3 tissue were homogenized with RIPA buffer and centrifuged at 12, 000×g for 15 min at 4°C. Then, the supernatants were collected and the protein concentration was measured using the BCA method and boiled in SDS loading buffer. After SDS/PAGE, the protein was transferred onto nitrocellulose membranes (Whatman) and then incubated with primary antibodies at 4℃ for overnight. Incubation of secondary antibodies was performed at room temperature for 1 h. The membranes were developed using the ECL detection system.

**1.6 | Immunohistochemistry**

The AD brain slices were soaked in xylene at 37°C overnight for dewaxing. Then, slices were washed with PBS containing 0.2% Tween 20 (PBST) for 3 x 5 min, incubated with 3% H_2_O_2_ in absolute ethanol for 15 min, 0.5% Triton X-100 for 30 min and nonspecific sites were blocked with 3% bovine serum albumin (BSA) for 30mins at room temperature. After incubation with primary antibodies at 4°C overnight, slices were washed with PBST for 3 x 5 min and immunoreaction was developed using the HistostainTM-SP kit and visualized with diaminobenzidine (brown color). Slices were then dehydrated with a graded ethanol and mounted with a coverslip. Images were obtained with a microscope (Olympus BX60, Tokyo, Japan).

**1.7 | Immunofluorescence**

The brain slices or primary neurons fixed in 4% paraformaldehyde for 15 min were permeabilized in 0.5% triton X-100 phosphate buffer for 30min and then incubated with phosphate buffer containing 0.1% triton X-100 and 3% BSA to block nonspecific binding. Primary antibody incubation was performed at 4℃ for overnight. After washing with PBS, samples were subsequently incubated with secondary antibodies at 37℃ for 1 hr and counterstained with DAPI. Images were acquired using Carl Zeiss LSM710 confocal microscope.

**1.8 | RT-qPCR**

Total RNA was isolated by TRIzol (Invitrogen, CA) and reversely transcribed using the PrimeScript RT Master Mix (Perfect Real Time). The produced cDNA was used for real-time PCR with primer sets: 5’-AAGCCCACCTGTAAGAA-3’ and 5’-ATCATCCCAATAATCCAC-3’ used for TRPC1, 5’-GCACGCTCAATGTCAACT-3’ and 5’-TGCACCACCTCGTACTTAT-3’ used for TRPC3, 5’-AGAAGAGCCAGAGCGAAGG-3’ and 5’-TGCCGCCTGTTGACGA-3’ used for TRPC4, 5’-CAAGGCTGAGTACGAGG-3’ and 5’-TTGGCTGAGCGACAAA-3’ used for TRPC5, 5’-TGGGACCCTACCGA-3’ and 5’-TTGATGACCACCGACT-3’ used for TRPC6, 5’-TGCTCAAGGGTGCG-3’ and 5’-CTGGATAGGGACAGGTAG-3’ used for TRPC7, 5’-TCTGAAGAGTCTACCGAAGC-3’ and 5’-GGTCCTCCACGCTGATA-3’ used for STIM1, 5’-CTCCATTCCACCCTACC-3’ and 5’-GGCACTTCCCATTGTTT-3’ used for STIM2, 5’-GCCTTCTCCACCGTCAT-3’ and 5’-AAGCGGGCAAACTCG-3’ used for ORAI1, 5’-TGGTAGCGATGGTGGAA-3’ and 5’-TTGACGGAGTTGAGGTTGT-3’ used for ORAI2, 5’-GGTGGGCTGGGTCAAGT-3’ and 5’-CGGTAGAAATGTAGGGCAAA-3’ used for ORAI3, 5’-GCACAGCGACGAGTACAAGATC-3’ and 5’-GAACAAGTTCCGCAGGGTG-3’ used for C/EBPβ and 5’-GTTGACATCCGTAAAGACC -3’ and 5’-GGAGCCAGGGCAGTAA -3’ used for β-actin.

**1.9 | Animals and Stereotaxic Surgery**

Wild-type male C57BL/6J mice (2-month-old, 110 ± 10g) were purchased from Experimental Animal Center of Wuhan University. TRPC1 knockout mice were a kind gift from Dr. Lutz Birnbaumer (Laboratory of neurobiology, National Institute of Environmental Health of Science, Research Triangle Park, NC 27709). Two-month-old male TRPC1 knockout mice of 129/SvEv genetic background and age-matched wild-type mice kept under standard laboratory conditions: 12-h light/dark cycle with accessible food and water. All animal studies were performed according to the “Policies on the Use of Animals and Human in Neuroscience Research” revised and approved by Society for neuroscience 1995 and by the Academic Review Board of Tongji Medical college, Huazhong University of Science and Technology. For stereotaxic surgery, mice were anesthetized with isoflurane and placed on a stereotaxic apparatus, and then, 1 μL AAV-syn-TRPC1-eGFP or AAV-syn-hTau-eGFP or the control AAV-syn-eGFP (with titers > 5*10^12^ IU/ml) were bilaterally injected into the hippocampal CA3 region (AP ±2.0, ML -1.5, DV -2.0) at a rate of 0.3 μL/min. the needle was kept for 10 min before withdrawal, the skin was sutured and mice were placed beside on a heater for recovery.

**1.10 | Golgi Staining and Spine Analysis**

Golgi staining was performed by using a FD Rapid Golgi Stain Kit (FD neurotechnology, PK401) as described before (Ye *et al.* 2019). Briefly, mice were anesthetized by isoflurane and intracardially perfused with normal saline for 10 minutes. After dissection, mouse brains were sequentially incubated with the staining buffer A, B, C, D, and E as described in the instruction. Then, sections were immersed in xylene (Sinopharm Chemical Reagent, 10023418) for 3 times (5 min each) and mounted with sealant. The images were obtained using Olympus BX60 (Tokyo). The spine density was analyzed using ImageJ software, which was presented as the number of spines per 10 μm of dendritic length.

**1.11 |Novel Object Recognition**

The mice were allowed to adapt to the box (50 cm x 50 cm x 50 cm plastic container) for 5 min 24 h before the test. A 5 min training phase was utilized for mice to familiarize themselves with object A and object B. The box was cleaned with 70% ethanol between each trail as well. Exactly 24 h after the training period, object B was replaced with a novel object C, and the mice were granted 5 min to explore both objects. The behavior was recorded by a video camera positioned above the box. The recognition times (T) for objects A, B, and C were recorded and calculated. The recognition index (TA / (TA + TB), TB / (TA + TB), and TC / (TA + TC)) and discrimination index (TC - TA) / (TA + TC) were analyzed.

**1.12 | Morris Water Maze**

The spatial learning and memory were assessed by Morris water maze (MWM) performed from 2:00 PM to 8:00 PM as described before (Yin *et al.* 2016a). During the learning period, mice were granted in the water maze to find a hidden platform under the milky water for consecutive 6 days, 3 trials per day with a 30 s interval. In each trial, the mice started from one of the four quadrants (exclude the target quadrant) facing the wall and ended when the mice climbed on the platform. The mice were guided onto the platform if the mice did not find the platform in 60 s. The latency and the swimming path were recorded by a video camera 1.5 m from the water surface. The spatial memory was measured 1 day after the last training. Mice were allowed to explore the water maze for 60 s with the platform removed. The time used to enter the target platform area, time spend in the target quadrant and target platform crossings were recorded by a digital video camera connected to a computer (Chengdu Taimeng Software Co. Lid, China).

**1.13 | Contextual Fear Conditioning**

The contextual fear conditioning test was performed one week after MWM as described before (Yin *et al.* 2016a). Briefly, 24 h before starting the test, the mice were habituated to the chamber for 3 min. During the training phase, the mice was subjected to 3 min of foot shock (0.8 mA, 2 s). The memory was carried out 24 h after the training by subjecting back into the chamber for 3 min, total freezing time during the 3 min was recorded for assessment of memory.

**1.14 | LTP Recording**

Mice were anesthetized using isoflurane and brains containing the dorsal hippocampus were cut into horizontal sections of 400 um thickness by a Leica VT1000s vibration microtome in ice-cold artificial cerebrospinal fluid (aCSF) consisting of (mM) 126 NaCl, 3 KCl, 1.25 NaH_2_PO_4_, 24 NaHCO_3_, 2 MgSO_4_, 2 CaCl_2_ and 10 glucose, equilibrated with 95% O_2_ and 5% CO_2_. Then slices were transferred in an interface chamber continuously filled with aCSF and maintained for 1h at room temperature. For excitatory postsynaptic potential (fEPSP) recordings, single slice was placed on a recoding chamber (MED-PA5455; Alpha MED Sciences, Kadoma, Japan) on the stage of an up-right microscope and kept submerged in aCSF (2ml/min, 30°C) at room at 24 °C. The fEPSP in CA1 stratum radiatum were recorded by stimulating the Schaeffer fibers from CA3 with a 0.1 MΩ tungsten monopolar electrode. LTP was induced through applying 3 trains of high-frequency stimulation (HFS, 100Hz, 1 s duration). LTP magnitude was calculated as the average (normalized to the baseline) of the data recorded 50-60 min after conditioning stimulation.

**1.15 | Human Samples**

Postmortem human brain samples were provided by Dr. Chao Ma of Human Brain bank, Chinese Academy of Medical Sciences and Peking Union Medical College, Beijing, China. AD was diagnosed according to the criteria of the consortium to Establish a Registry for AD and the National Institute on Aging. Information of human samples were listed in table 2.

**1.16 | Statistical Analyses**

Data were expressed as mean ± SEM or mean ± SD, and analyzed by GraphPad Prism 8 statistical software (GraphPad Prism). Student’s t-test was used for the comparison between two groups, the one-way ANOVA followed by Dunnett’s post hoc test or Tukey’s post hoc analysis was used to analysis the data among three, four, or six groups. The statistical significance was assessed at *p* < 0.05.
